# Episodic Memory Assessment and Remediation in Normal and Pathological Aging Using Virtual Reality: A Mini Review

**DOI:** 10.3389/fpsyg.2019.00173

**Published:** 2019-02-06

**Authors:** Valentina La Corte, Marco Sperduti, Kouloud Abichou, Pascale Piolino

**Affiliations:** ^1^Memory and Cognition Laboratory, Institute of Psychology, Paris Descartes University, Paris, France; ^2^Center for Psychiatry and Neuroscience, INSERM U894, Paris, France; ^3^Institute of Memory and Alzheimer’s Disease, Department of Neurology, Pitié-Salpêtrière Hospital, Paris, France; ^4^University Institute of France, Paris, France

**Keywords:** episodic memory, virtual reality, assessment, cognitive remediation, aging, Alzheimer’s disease

## Abstract

Life expectancy is constantly increasing in developed countries. Unfortunately, a longer life does not always correspond to a healthier life, as even normal aging is associated with cognitive decline and increased risk factors for neurodegenerative diseases. Episodic memory (EM) is one of the most vulnerable cognitive functions in aging, and its decline is the hallmark of typical Alzheimer’s disease. This memory system is defined as the ability to acquire and recollect personally experienced episodes associated with a specific affective, spatial, and temporal context. However, most of the neuropsychological and experimental tasks currently employed to assess EM consist in learning simple material (e.g., list of words) in highly stereotyped contexts. In the same vein, classical paper-and-pencil or numeric remediation tools have shown their limitations in the transfer of acquired skills to daily life. Virtual reality (VR), thanks to its immersive properties, and the possibility of delivering realistic and complex scenarios, seems a promising tool to address the limitations of the assessment and remediation of EM. Here, we review existing studies employing VR in normal and pathological aging to assess and reeducate EM. Overall, we show that VR has been mainly used via non-immersive systems. Further studies should, therefore, test the impact of different degrees of immersion. Moreover, there is a lack of VR remediation tools specifically targeting EM. We propose that future studies should fill this gap, addressing in particular the adaptivity of VR remediation protocols.

## Age-Related Cognitive Decline: the Crucial Role of Episodic Memory

Given the global aging of the population and its associated cognitive decline, designing sensitive diagnostic tools for early symptom detection, and effective intervention to attenuate age-related cognitive decline is a global societal priority. One of the first cognitive functions impacted by aging is episodic memory (EM). EM is defined as the ability to acquire and recollect personally experienced episodes (what) associated with a specific spatial (where), and temporal (when) context (Tulving, 1972; Tulving and Murray, 1985). This type of memory is based on associative processes known as “binding” that connect the contextual features of the specific events. EM impairment is also considered as the earliest clinical sign of typical Alzheimer’s disease (AD) (Epelbaum et al., 2018), preceding that of other cognitive functions (Grober et al., 2008; Derby et al., 2013). Rather than offering a complete measure of EM components, most clinical memory tests generally measure only one aspect in isolation (e.g., what). To overcome these limitations researchers have relied on advances in virtual reality (VR).

Virtual reality allows creating immersive and interactive multimodal environments. VR systems can vary in their degree of immersion and interaction which, in turn, modulates the subjective feeling of presence, defined as the feeling of “being there” (e.g., Cipresso et al., 2018). For the purpose of this review, we will classify VR systems on two orthogonal axes: immersion and interaction. On the first axis, there are at one extreme non-immersive systems (NIS) that present the VR environment on desktop computers, and at the opposite pole, fully immersive systems (FIS) that employ head-mounted displays (HDM) with head-movement tracking systems allowing sensory-motor contingency. On the second axis, at the lowest level, there are systems where participants are passive (PS), and at the highest level participants can complexly interact (IS) with the environment thanks to different kinds of devices (e.g., steering wheel, cyber-gloves, posture tracking sensors). For the sake of clarity, we will employ throughout the manuscript this terminology, while recognizing that VR systems can present intermediate levels on both axes.

At the theoretical level, the use of VR in memory studies addresses the pivotal role of self-experience and bodily representation, raised by the embodied cognition framework (Barsalou, 2008; Shapiro, 2011), in supporting EM (Bergouignan et al., 2014; Repetto et al., 2016; Tuena et al., 2017; Blanke et al., 2018). Repetto et al. (2016) pointed out three main VR features that may impact EM: (1) VR enables situations to be experienced from an egocentric point of view; (2) VR allows active exploration of the environment; and (3) VR provides environmental enrichment by using flexible scenarios with different degrees of complexity. Moreover, by employing VR researchers can reproduce complex real-like scenarios while maintaining high experimental control over stimuli presentation. This enables the complexity of EM to be investigated, in particular, its central feature of relying on contextual associations. Thus, one of the most important advantages of using VR consists in the evaluation of the binding process (Abichou et al., 2017; Plancher and Piolino, 2017). In this mini review, we give an overview of recent representative studies employing VR to assess and remediate EM in healthy and pathological aging. We will conclude by highlighting the limitations of existing work and proposing a roadmap for future studies.

## Episodic Memory Assessment in Normal and Pathological Aging Via Virtual Reality

### Assessment in Healthy Aging

In a first pilot study, Plancher et al. (2008) employed NIS VR (the environment was projected on a 85 cm × 110 cm screen) to investigate the effects of age (young vs. older adults), encoding (intentional vs. incidental), and exploration (active vs. passive) on the main aspects of EM. In the passive condition (PS) subjects were immersed as passengers of a virtual car, while in the active one (IS) they drove the car with a steering-wheel and a gas pedal. Participants navigated in a virtual town, encountered different events (e.g., a car accident), and at the end of the navigation they carried out a recall session assessing memory for items and contextual information. The main results showed that aging was associated with diminished contextual memory, but not factual memory, and that the only significant difference was in intentional encoding. However, no effect of the type of exploration (active or passive) was observed. In a following study, employing the same VR system, the authors investigated the associative mechanism of EM by computing different binding scores according to the richness of the contextual information recalled (Plancher et al., 2010). The encoding phase (incidental or intentional) occurred during active navigation of the environment. They reported that older participants were selectively impaired in higher-order binding (e.g., associating several items of contextual information), in particular in recalling the spatio-temporal context in the intentional encoding condition. Moreover, they showed that memory performances assessed using VR were more reliably associated with general cognitive functioning and subjective memory complaints, compared with standard neuropsychological tools.

Other studies have compared the effect of passive vs. active navigation in a virtual environment in modulating age-related differences in memory performances. Sauzéon et al. (2016) used a NIS VR (the environment was projected on a 200 cm × 188 cm screen) based on the California Verbal Learning Test (Delis et al., 1987) to investigate memory for everyday objects (Sauzéon et al., 2012). They reported that active navigation, performed using the keyboard and the mouse, increased recognition hits compared with passive navigation in both young and elderly participants. Furthermore, active navigation decreased false recognitions in young adults, but an opposite effect was observed in older participants. The association between false recognition and age was totally mediated by executive function performances. This is consistent with the idea of an additional burden induced by real-life conditions (e.g., active navigation) on source monitoring in aging.

Jebara et al. (2014), employing a NIS environment (projected on a large screen covering 66° of the visual field), went a step further, studying the influence of different degrees of interaction on EM encoding: passive navigation (subjects were passengers of a virtual car), itinerary control (subjects chose the itinerary, but did not physically drive the car), low navigation control (subjects controlled the displacement of the car on rails by a gas pedal on a fixed itinerary), or high navigation control (subjects drove the car with the steering-wheel and a gas pedal on a fixed itinerary). The task was to memorize as many events as possible along with contextual features. Results showed a general age-related decline for immediate and delayed feature binding. Compared to passive and high navigation control conditions, and regardless of age groups, feature binding was enhanced in the low navigation and in the itinerary control conditions. Interestingly, memory performances following the high navigation condition depended strongly on executive functioning.

The aforementioned studies employed VR to simulate the encoding of real-life events and tested memory performance outside VR. Other studies, on the contrary, employed realistic virtual scenarios to test memory, while performing encoding in more classical conditions (e.g., memorizing a shopping list). Parsons and Barnett (2017) recently tested the Virtual Environment Grocery Store (VEGS) task as a measure of EM function in the elderly. VEGS comprises a NIS simulation of a grocery store in which participants have to carry out different activities (IS) constructed to measure both prospective memory (e.g., return to the pharmacist when they hear their number), and retrospective EM (e.g., finding and selecting items from a shopping list that has been previously learned). VEGS memory scores significantly correlated with scores of standard neuropsychological episodic verbal memory tests, but not with those of executive functions, supporting the construct validity of this tool.

The Virtual Shop is a new FIS-IS interactive environment in which participants have to select a list of items, previously memorized outside VR, during navigation in a convenience store (Corriveau Lecavalier et al., 2018). In their first study, the authors compared the performances of young and old participants, reporting that older adults performed less well. In the second study, they tested older adults with subjective cognitive complaints on the same task and standard measures of memory, executive functions, and a subjective measure of memory difficulties in daily life, comprising questions referring to shopping activities (e.g., How often do you forget to buy something you intended to buy?). They reported that performance in the Virtual Shop was correlated with the measure of memory in daily life and a standard measure of memory. In addition, their findings indicate that the use of a fully immersive task is feasible in older adults: it elicits presence, it is engaging, and provokes limited symptoms of cybersickness (Ouellet et al., 2018).

Another recent study designed a VR NIS-PS task to reproduce the CVLT rendered by a stereoscopic HD projection system (3D), in young and older adults using a kitchen scene (Pflueger et al., 2018). The results showed that age-related learning and performance decrements were mainly evident in the standard CVLT but not in the VR-memory examination. The authors concluded that performances in VR tasks might provide a more accurate “age fair” estimation of everyday life than standard neuropsychological tools.

### Assessment in Pathological Aging

So far, the functioning of EM in AD and amnestic mild cognitive impairment (aMCI), such as memory for items, has been mainly evaluated with verbal material, indicating deficits of free and cued recalls, and recognition. There are only a few studies employing VR to assess EM in neurodegenerative diseases (García-Betances et al., 2015).

Widmann et al. (2012) used a photorealistic NIS virtual reality model of a city (projected on a 200 cm × 300 cm screen) with passive navigation (i.e., NIS-PS) to assess verbal and spatial EM, comparing it to standardized neuropsychological tests in healthy elderly control participants and patients with mild AD. Participants were instructed that they were going on a shopping trip through the jewelry district of a large city. Then they were asked to read and remember the names of specific shops. The authors reported that the increased complexity of learning in a virtual environment brings to the fore the impairment of free memory recall in patients in a more clear-cut way than that achieved with classical list learning tests. Thus classical neuropsychological tasks may underestimate memory capacity in daily life situations. The authors concluded that learning verbal material in a realistic virtual environment is comparable to classic list learning in healthy elderly individuals but is more severely impaired in very mild AD patients.

In another study, Plancher et al. (2012) tested healthy older adults, patients with aMCI, and patients with AD with a NIS-IS or PS virtual task similar to those employed in the aforementioned studies (Plancher et al., 2008, 2010). They found that AD patients’ performances were poorer than those of aMCI patients, and even more so than those of healthy elderly. Spatial allocentric memory was found to be particularly useful for distinguishing aMCI patients from healthy older adults. Active exploration, compared to passive exploration, yielded enhanced recall of central and allocentric spatial information, as well as binding in all groups. This led aMCI patients to achieve better performances on immediate temporal memory tasks. Finally, the patients’ memory complaints were more strongly correlated with their performances in the virtual test than with those achieved in classical memory tests.

In summary, some of the previous studies in healthy and pathological aging investigated the effect of the degree of interaction of the VR system on EM. The results are not consensual, with some studies reporting no differences between an active and passive condition (Plancher et al., 2008), while others reported a beneficial or detrimental effect for correct and false recognition, respectively (Sauzéon et al., 2016). Still other studies reported an enhancement or decrease in performances depending on the type of immersion (Jebara et al., 2014), with the highest benefit being obtained with degrees of interaction that do not excessively tax cognitive resources. Indeed, the association between the degree of interaction and memory performance has been shown to depend on the executive performances. These findings suggest that the interaction should be tailored, particularly when using VR as a cognitive remediation tool, to the cognitive abilities of the participants. On the contrary, with the exception of one study (Corriveau Lecavalier et al., 2018), the aforementioned research exclusively employed NIS. Thus, even if theoretically sound, the impact of different degrees of immersion on EM has not been directly tested. Although the study of Corriveau Lecavalier et al. (2018) suggests the feasibility of using FIS to assess EM in the elderly, they did not compare it to a NIS to assess the modulatory role of immersion on EM. These studies, however, highlight the benefit of using VR in assessing EM in both healthy and pathological aging, since the performances obtained with VR tasks correlated better with daily life difficulties and subjective memory complaints. Moreover, VR evidenced EM impairment in mild AD more straightforwardly.

## Remediation in Normal and Pathological Aging Via Virtual Reality

Recent advances in VR technology have improved applications for cognitive rehabilitation (Larson et al., 2014). Several studies have shown the efficiency of VR in cognitive remediation (Rizzo et al., 2004), and its transfer to real life thanks to a multisensory and immersive environment. Within this context, Clemenson and Stark (2015) demonstrated that training (via NIS-IS) naive video gamers on a complex 3D strategic game, but not on a simple 2D fighting game, improved EM and spatial memory in tasks known to be related to hippocampal activity in young subjects (e.g., item discrimination, virtual water-maze). On similar lines, West et al. (2017) showed that video-game training (via NIS-IS) using a specific 3D video-game (Super Mario 64 with a Nintendo Wii console system) can have a positive effect on the hippocampal memory system in older adults.

Although these studies (Clemenson and Stark, 2015; West et al., 2017) used well-known video-games (e.g., Super Mario), instead of realistic environments, their findings support the view that EM can be enhanced in older adults by means of enriched 3D scenarios. In a more ecological context, Gamito et al. (2018) designed a set of NIS VR exercises that mimic activities of daily life by which elderly subjects can train different cognitive domains. They reported significant performance increases for some of the neuropsychological measures: visual memory, attention, and cognitive flexibility.

Other VR systems have been proposed to enhance cognition in both the elderly and in pathological aging. A systematic review by Jekel et al. (2015) suggested that various VR applications for instrumental activities of daily living assessment could also be employed for cognitive training in MCI patients (Werner et al., 2009). For instance, Zygouris et al. (2015) developed the Virtual Supermarket (VSM) NIS-IS cognitive training, designed to mimic daily shopping in a supermarket, aiming at improving multiple aspects of the cognitive functioning that are necessary for autonomous living in aging (e.g., the patient is asked to buy items displayed on a shopping list). Preliminary results suggested that monitoring longitudinal performance on the VSM training could provide useful diagnostic information to detect MCI (Zygouris et al., 2017). In the same vein, in the GRADYS FIS-IS software, which combines simulation of everyday life activities and different levels of difficulty, the participants are able to control their immersion and interaction with VR using a headset (Oculus Rift) and a control pad (Zając-Lamparska et al., 2017). The above-mentioned studies focused mostly on navigation skills, cognitive functionality, and other instrumental activities of daily living, not specifically on EM. VR has also been used for training patients with Alzheimer’s disease in everyday activities. Foloppe et al. (2018) developed a NIS-IS VR platform for training AD patients in everyday cooking activities based on errorless learning, vanishing-cue, and virtual reality techniques. A first case study showed that the AD patients were able to relearn some cooking activities using VR and transfer this learning to real life.

## Conclusion and Perspectives

We have presented a glimpse of VR applications for the assessment and training of EM in normal and pathological aging. It is difficult to unambiguously conclude on which features of VR are the most important in assessing and stimulating EM, since although some studies manipulated the degree of interaction of the VR system, only one study employed FIS. The immersion dimension of VR, surprisingly, has not been studied extensively. Future studies should compare VR systems by systematically varying the degree of immersion and interactivity to determine the best setting for studying EM. Moreover, as emerges from the literature reviewed above, the best technological features (e.g., interaction) of a given system may partially depend on the population that is being tested. This is particularly important in designing VR remediation tools. A central aspect of VR experience that has been neglected in studies on EM is presence. This is surprising given the importance of presence in the VR literature *per se*, its relation with EM performance in VR (Nash et al., 2000), and recent empirical findings on the link between presence and EM in ecological settings (Makowski et al., 2017). One of the possible reasons for this gap is that presence is often assessed via subjective reports, with no consensual objective measures. Future studies should address this issue.

Moreover, to date, research on VR-based neuropsychological assessments has been largely exploratory and often lacking in psychometric rigor (Parsons and Barnett, 2017). Further research should provide normative data on the different tasks in order to introduce them in clinical neuropsychological practice.

Concerning rehabilitation applications, preliminary results point out the feasibility of VR training in healthy elderly as well as in pathological populations. Nevertheless, apart from two studies using 3D video-games, none of the VR studies based on simulation of a naturalistic environment and daily life activities specifically targeted EM. Moreover, even if research in other cognitive domains (e.g., cognitive control) has shown that the adaptivity of the training plays a pivotal role in its efficacy (Anguera and Gazzaley, 2015), no research has addressed this feature of VR training specifically targeting EM. A vital feature that could make an important impact on future VR clinical applications could be the development of virtual assessment and training tools that are flexible and adaptable to the individual’s cognitive profile.

## Author Contributions

VLC, MS, and KA collected the materials and resources needed for this review. VLC and MS conceived and wrote the paper. PP provided suggestions on the structure of the paper and revised each draft. VLC, MS, and PP approved the final version of the manuscript.

## Conflict of Interest Statement

The authors declare that the research was conducted in the absence of any commercial or financial relationships that could be construed as a potential conflict of interest.
